# Effect of tourniquet use on blood loss, pain, functional recovery, and complications in robot-assisted total knee arthroplasty: a prospective, double-blinded, randomized controlled trial

**DOI:** 10.1186/s13018-022-02992-y

**Published:** 2022-02-21

**Authors:** Ya-hao Lai, Hong Xu, Qiang Su, Xu-feng Wan, Ming-cheng Yuan, Zong-ke Zhou

**Affiliations:** Department of Orthopaedics, West China Hospital of Sichuan University, No.37, Guoxue Road, Wuhou District, Chengdu, 610041 Sichuan People’s Republic of China

**Keywords:** Robot-assisted total knee arthroplasty, Tourniquet, Blood loss, Pain, Functional recovery

## Abstract

**Background:**

Robot-assisted total knee arthroplasty (TKA) has been largely studied to confirm its advantages in terms of accurate component positioning, microembolus formation, less blood loss, and so on, but is currently usually performed under tourniquet due to its longer operative time than conventional TKA. The aim of this study was to estimate the effects of tourniquet use in robot-assisted TKA on blood loss, pain, functional recovery, and complications.

**Methods:**

Patients scheduled for robot-assisted TKA were prospectively randomized into a tourniquet or non-tourniquet group (each *n* = 14). The primary outcome measure was blood loss. The secondary outcome measures were operation time; visual analog scale (VAS) pain scores; time to achieve the first straight-leg raise; swelling of the thigh, knee, and calf; range of motion; Hospital for Special Surgery score; length of stay; and postoperative complications.

**Results:**

There was no significant difference in total blood loss between the tourniquet and non-tourniquet groups (738.57 ± 276.158 vs. 866.85 ± 243.422 ml, *P* = 0.061). The tourniquet group showed significantly lower intraoperative blood loss (*P* < 0.001), but higher hidden blood loss (*P* = 0.002). The non-tourniquet group showed better knee range of motion on postoperative days (PODs) 1–3 (all *P* < 0.001), less thigh swelling on PODs 2 and 3 (*P* < 0.05), earlier straight-leg raising (*P* = 0.044), and shorter length of stay (*P* = 0.044). Thigh pain VAS score at 1 month after surgery was significantly greater in the tourniquet group (*P* < 0.001), as was knee pain during activity and at rest on PODs 2–3 (all *P* < 0.05). The tourniquet group also showed a significantly higher rate of tension blisters (28.8% vs. 7.1%, *P* = 0.038).

**Conclusions:**

Tourniquet use during robot-assisted TKA does not reduce total blood loss, and it appears to increase postoperative pain, aggravate muscle injury, and prolong postoperative recovery.

*Trial registration* ChiCTR, ChiCTR2100041800. Registered 5 January 2021, http://www.chictr.org.cn/index.aspx.

## Introduction

An estimated 30.8 million adults in the USA alone and 300 million individuals worldwide are living with osteoarthritis [1]. Total knee arthroplasty (TKA) has become the gold-standard treatment for patients with severe knee osteoarthritis whose pain was not controlled by other interventions [2]. The patients who need surgery with effective outcomes highlight the need for measures that increase the durability and longevity of prostheses.

Judicious choice of prosthetic components and careful positioning of the implant can prolong prosthesis life and improve patient satisfaction [3–6]. Robot-assisted TKA can allow more accurate component positioning [7, 8], particularly in difficult situations, such as when patients are obese or have suffered post-traumatic deformity or when patients are undergoing revision knee arthroplasty [9, 10]. In addition, robot-assisted TKA can reduce blood loss by avoiding the need to insert instrumentation into the intramedullary canal [11], which also reduces risk of operator error [12] and microembolus formation [13]. Robot-assisted TKA may eliminate the requirement for a bloodless visual field that applies to the conventional procedure [14]. The computer system can automatically select the osteotomy surface.

Nevertheless, robot-assisted TKA is associated with longer operation time [15], especially before the clinician has gained experience, since the procedure involves a long learning curve [16]. This longer operation time can lead to higher intraoperative blood loss, so robot-assisted TKA is typically performed with a tourniquet [17].

This view seems to be contrary to that of studies on non-robotic TKA. Most studies about tourniquet use in conventional TKA believe that tourniquet use cannot reduce the total blood loss and will lead to delayed recovery [5, 17–21]. However, the study of tourniquet use in robot-assisted TKA has not been reported. Therefore, we conducted a prospective trial in which patients receiving robot-assisted TKA were randomized into tourniquet and non-tourniquet groups, and we examined the influence of the tourniquet on blood loss, pain, functional recovery, and complications.

The aim of this study was to estimate the effects of tourniquet use in robot-assisted TKA on blood loss, pain, functional recovery, and complications.

## Methods

### Patients

This study obtained the approval of the ethics committee and institutional review board of our hospital, and informed patient consent for each enrolled patient was also obtained. The study was designed as a prospective, double-blinded, randomized controlled trial. Between January 2021 and March 2021, thirty eligible patients were enrolled in this study. Two of them were excluded because the degree of severe osteoarthritis that had to be treated with TKA was not reached.

Inclusion criteria were as follows: (1) age between 18 and 80 years, (2) diagnosis of severe knee osteoarthritis that failed to be treated conservatively, (3) investigator-assessed requirement for primary unilateral TKA, and (4) full patient understanding of the benefits and risks of this study and written informed consent. Patients were excluded if they had (1) coagulation disorders, based on preoperative routine monitoring of activated partial thromboplastin time (APTT), prothrombin time (PT), fibrinogen (FIB), thrombin time (TT), (2) body mass index > 40 kg/m^2^, or (3) history of lower limb fracture.

Patients were randomized into a tourniquet group and non-tourniquet group according to a computerized random sequence generator. The researcher collecting data were uninvolved in study procedures and were blinded to group allocation.

### Perioperative management

The same surgeon performed all the operations, and patients received combined intravenous and inhalation anesthesia with a standardized regimen. A pneumatic tourniquet was applied to the tourniquet group at a pressure of 240 mmHg throughout the surgery. The skin under the tourniquet was covered with a single layer of cast padding on a single layer of stockinette. The tourniquet was inflated before the skin incision and deflated after suturing. We relied on preoperative computed tomography (CT) of the hip, knee, and ankle to help the clinician decide the optimal prosthetic orientation in the robot-assisted system YUANHUA-TKA (Yuanhua Intelligent Technology, Shenzhen, China). Procedures were performed with patients in the supine position, with the tibia and foot in a mobile boot. Femoral and tibial registration pins were used to register the leg position in the system at the start of the operation. We utilized a midline skin incision with a medial parapatellar approach for every patient. Bone cuts were made with robotic arm assistance based on the preoperative system plan. The surgeon could modify bone resection by using a dynamic tracking system to track alignment, flexion, extension gaps, and range of movement.

All patients received the same perioperative treatment strategies: tranexamic acid (TXA), pain management, thrombosis prevention, and functional rehabilitation. Celecoxib (200 mg) was administered twice a day from admission to 14 days after discharge. All patients received 2 g of intravenous TXA at 30 min before the incision and 1 g of intravenous TXA at 3 and 6 h after the surgery. All patients received 5 mg dexamethasone intravenously before TXA. For thrombosis prophylaxis, we administered 0.2 ml of low molecular weight heparin (enoxaparin) at 8 h after the procedure, which was increased to 0.4 ml per day from the next day until discharge. Subsequently, apixaban was continued for two weeks.

Patients were discharged when (1) pain control was judged to be adequate, defined as a visual analogue scale (VAS) score < 3, (2) the patient could actively flex the knee to at least 90°, and (3) the patient could walk independently with a walking aid.

### Primary outcomes

The primary outcome was blood loss, defined to include total blood loss, intraoperative blood loss, drainage volume, and hidden blood loss. The total blood loss volume of each patient was calculated according to Nadler's formula [22]. Gross's formula was then used to calculate total blood loss based on total blood volume and hematocrit drop [23]. Intraoperative blood loss was estimated from the increase in gauze weight and the volume (excluding saline) in the negative pressure aspirator bottle. The drainage tube was removed at 24 h after the operation, and the drainage volume was recorded. Postoperative blood loss was calculated from the drainage volume and dressing weight. Hidden blood loss was defined as total blood loss minus intraoperative and postoperative blood loss.

### Secondary outcomes

Secondary outcomes were operation time; VAS scores; time to achieve the first straight-leg raise; swelling of thigh, knee and calf; range of motion; Hospital for Special Surgery (HSS) score; length of stay; and postoperative complications. These outcomes were assessed at admission, every morning on postoperative days (PODs) 1–3, on the day of discharge, and at one and three months postoperatively. On the mornings of PODs 1–3, VAS scores were used to assess thigh pain, knee pain at rest, and knee pain during activity (when knees were flexed to the maximum extent).

Postoperative complications were recorded and included tension blisters, ecchymosis, superficial infection, numbness of lower limbs, and deep vein thrombosis (DVT). DVT was monitored through a bilateral lower extremity deep venous color Doppler ultrasound examination on POD 1 and at 1 month postoperatively.

After returning to the ward from the postanesthesia care unit, the patient was told to make his or her best effort to perform straight-leg raising, and the time needed for this was recorded by a nurse. Swelling in the lower limbs was assessed by measuring circumferences of the thigh (10 cm above the patella midline), knee (patella midline), and calf (10 cm below the patella midline).

All patients received physical therapy from the same rehabilitation physician on POD 1. On PODs 1–3, range of motion was measured using a goniometer based on how far the patient was able to flex the knee.

### Statistical analysis

The minimal sample size was estimated based on a previously reported mean blood loss of 613 ml for robot-assisted TKA [11] and our definition of 200 ml as the smallest clinically meaningful reduction in blood loss due to tourniquet use. These considerations, combined with a medium effect size of 0.5, power of 0.8, and alpha error of 0.2, meant that at least 28 patients had to be enrolled in the study.

Data were reported as mean ± standard deviation (SD) if they were normally distributed according to the Shapiro–Wilk test. Otherwise, data were reported as the median (25th percentile, 75th percentile). Inter-group differences in continuous variables were assessed for significance using Student’s *t* test or the Mann–Whitney test, depending on the type of data and whether they were normally distributed. Differences in categorical data were assessed using Fisher's exact test. Differences associated with *P* < 0.05 were considered significant. All analyses were performed in SPSS 26.0 (IBM, Armonk, NY, USA).

## Results

### Participants

**Fig. 1:**
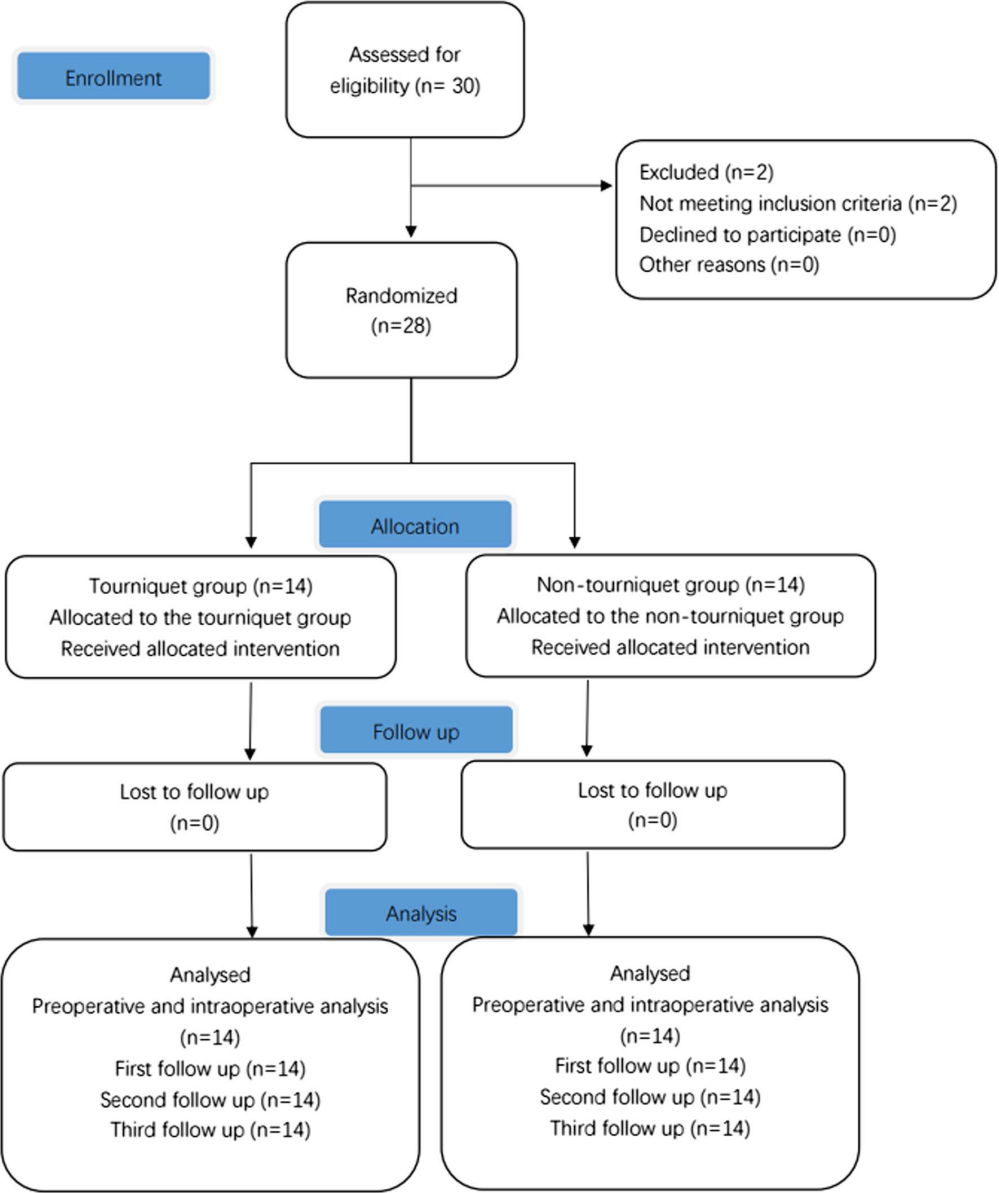
Flow diagram of patient enrollment and analysis

Of the total 30 potentially eligible patients treated during the recruitment period, 2 were excluded because they did not meet the inclusion criteria. In total, 28 patients were randomly and equally divided into two groups. There were no significant differences in clinico-demographic characteristics between the two groups (Table 1). No patients were lost to follow-up (Fig. 1).

**Table 1:** Preoperative characteristics of patients who underwent robot-assisted TKA with or without a tourniquet

Characteristic	Tourniquet group (*n* = 14)	Non-tourniquet group (*n* = 14)	P*
Sex			0.532
Male	4	3	
Female	10	11	
Age (years)	64.00 (6.565)	65.57 (7.700)	0.400
Body mass index (kg/m^2^)	27.76 (3.266)	27.40 (3.476)	0.417
Knee range of motion (°)	90 (90, 100)	100 (90, 100)	0.179
Hospital for Special Surgery score	59.33 (5.352)	53.73 (7.520)	0.068
Hemoglobin (g/L)	138.57 (12.800)	137.97 (13.011)	0.858
Hematocrit (%)	0.43(0.037)	0.42 (0.032)	0.534
Visual analog scale score			
Thigh pain	0.03 (0.183)	0.00 (0.000)	0.321
Knee rest pain	3 (0, 5)	3 (2, 5)	0.418
Knee active pain	7 (6, 8)	7 (6, 8)	0.730
Circumference (cm)			
Thigh	40.22 (3.545)	39.95 (3.770)	0.779
Knee	36.97 (2.300)	36.80 (2.568)	0.792
Calf	36.60 (2.654)	32.60 (2.881)	0.167

Values are *n*, mean (standard deviation) or median (interquartile range), unless otherwise noted

*Based on Student’s *t* test or the Mann–Whitney test (continuous variables) or Fisher’s exact test (categorical variables)

### Blood loss

Between the tourniquet and non-tourniquet groups there was no significant difference in total blood loss (738.57 ± 276.158 vs. 866.85 ± 243.422 ml, *P* = 0.061) or drainage volume [145 (100, 180) vs. 95 (23.75, 210) ml, *P* = 0.071]. The tourniquet group showed significantly lower intraoperative blood loss (134.17 ± 57.191 vs. 308.67 ± 63.122 ml, *P* < 0.001), but significantly higher hidden blood loss (511.15 ± 183.322 vs. 366.00 ± 171.281 ml, *P* = 0.002). No patient required blood transfusion at any time (Table 2).

**Table 2:** Perioperative blood loss, transfusion, and surgical time in the two patient groups

Variable	Tourniquet group (*n* = 14)	Non-tourniquet group (*n* = 14)	P*
Surgical time (min)	95 (80, 101.25)	107 (93.75, 135)	0.005
Total blood loss (ml)	738.57 (276.158)	866.85 (243.422)	0.061
Intraoperative blood loss (ml)	134.17 (57.191)	308.67 (63.122)	< 0.001
Drainage volume (ml)	145 (100, 180)	95 (23.75, 210)	0.071
Hidden blood loss (ml)	511.15 (183.322)	366.00 (171.281)	0.002
Transfusion (*n*)	0	0	N/A

Values are *n*, mean (standard deviation) or median (interquartile range), unless otherwise noted

^*^ Based on Student’s *t* test or the Mann–Whitney test (continuous variables) or Fisher’s exact test (categorical variables)

### Secondary outcomes

**Fig. 2:**
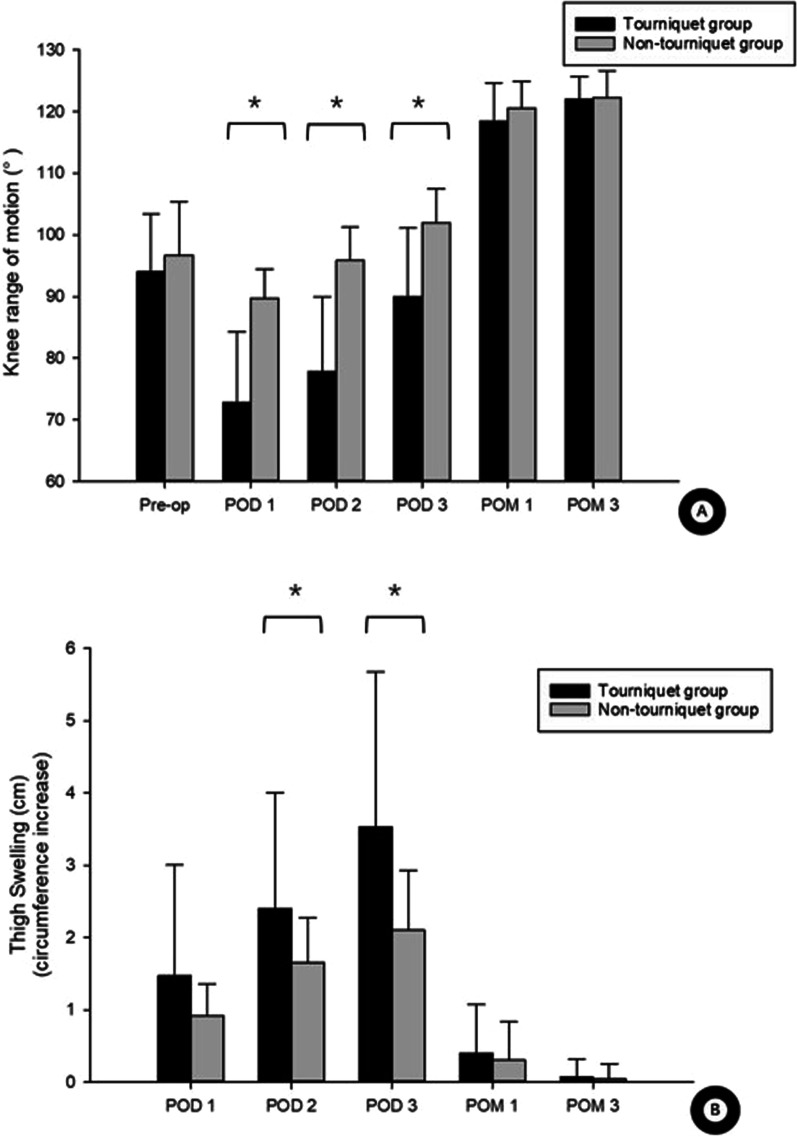
Differences in **A** knee range of motion and **B** thigh swelling between patients who underwent robot-assisted TKA with or without a tourniquet. *P < 0.05. *POD* postoperative day, *POM* postoperative month, *Pre-op* preoperative

Although the non-tourniquet group underwent surgery for a significantly longer time (117.57 ± 33.184 vs. 96.77 ± 21.379 min, *P* = 0.005), they achieved better knee range of motion on PODs 1–3 (Fig. 2A). However, the two groups did not differ significantly in knee range of motion at later time points or in knee functional recovery based on HSS score.

**Fig. 3:**
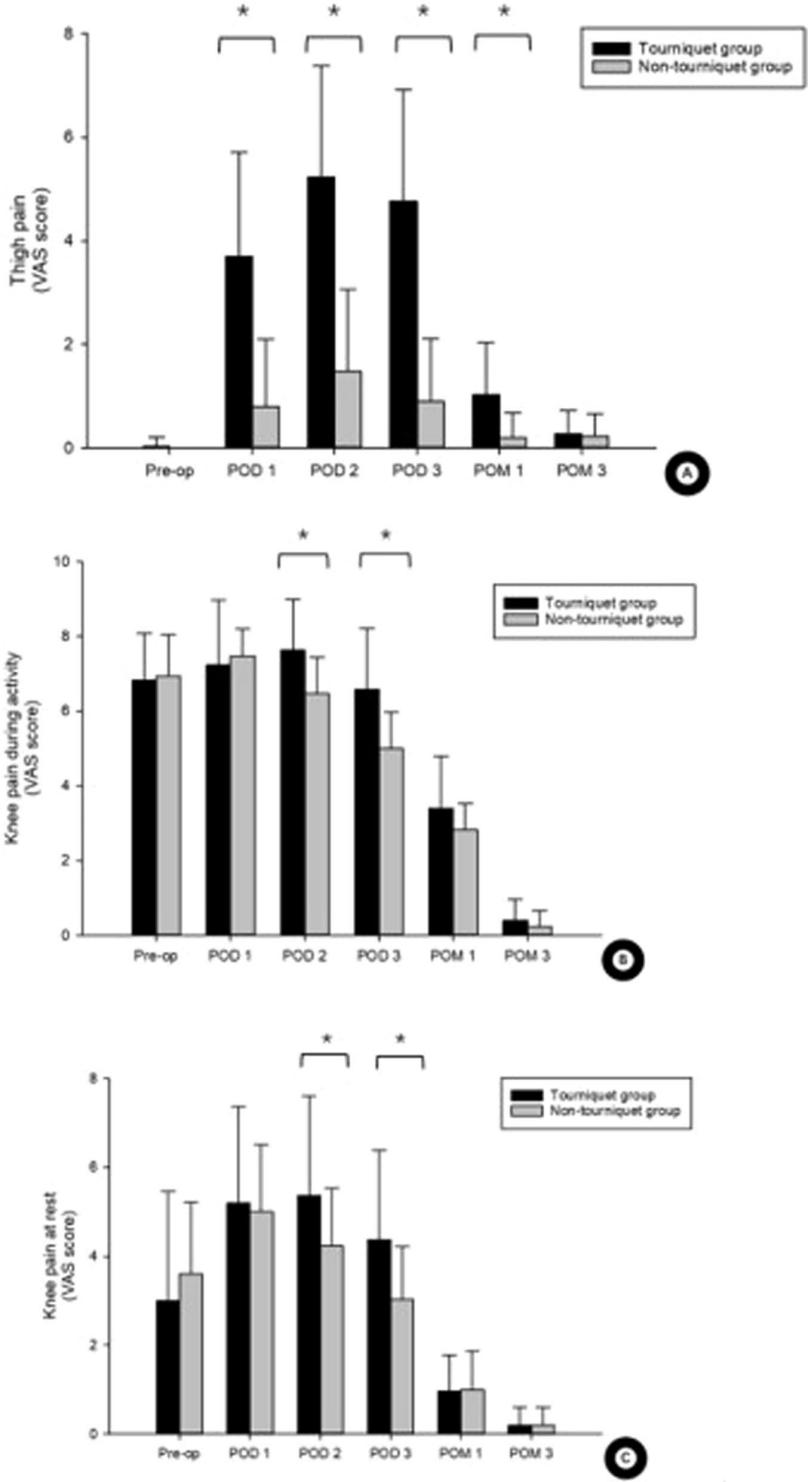
Differences in **A** thigh pain, **B** knee pain during activity, and **C** knee pain at rest. *P < 0.05. VAS, visual analog scale. *Pre-op* preoperative, *POD* postoperative day, *POM* postoperative month

On PODs 2–3, thigh swelling was more obvious in the tourniquet group than in the non-tourniquet group (Fig. 2B). At one month after surgery, thigh pain VAS scores were significantly higher in the tourniquet group (Fig. 3A). On PODs 2 and 3, but not at later time points, the tourniquet group reported more severe knee pain than the non-tourniquet group during activity and at rest (Fig. 3B, C).

A significantly higher number of patients in the tourniquet group (*n* = 4) experienced tension blisters than in the non-tourniquet group (*n* = 1) (*P* = 0.038). All blisters resolved with medical treatment including enhanced disinfection, pain relief, antipruritic, and other symptomatic treatments. The two groups did not differ significantly in rates of ecchymosis, numbness of lower limbs, superficial infection, or DVT (Table 3).

**Table 3:** Functional outcomes and postoperative complications

Variable	Tourniquet group (*n* = 14)	Non-tourniquet group (*n* = 14)	P*
Time to achieve the first straight-leg raise (h)	70 (18.75, 96)	13.5 (7, 17.25)	< 0.001
Length of stay (d)	6.5 (6, 7)	6 (6, 7)	0.044
Tension blisters	4	1	0.038
Ecchymosis	2	1	0.112
Numbness of lower limbs	1	0	0.237
Superficial infection	0	0	NA
Deep vein thrombosis	2	1	0.158

Values are *n* or median (interquartile range), unless otherwise noted

*NA* not applicable

The non-tourniquet group took significantly less time to achieve the first straight-leg raise, and their length of stay was shorter [6 (6–7) days, *P* = 0.044].

## Discussion

In what appears to be the first study to explore whether the tourniquet is beneficial to patients undergoing robot-assisted TKA, our data suggest that using a tourniquet in robot-assisted TKA does not decrease total blood loss and instead leads to more severe pain, slower functional recovery, longer hospital stay, and more frequent complications.

Although many clinicians remain skeptical about robot-assisted TKA owing to the substantive set-up costs and limited long-term evidence comparing clinical and functional outcomes to conventional manual TKA, robot-assisted TKA has gathered momentum as an avenue for improving the accuracy of implant positioning and reducing outliers in limb alignment [24]. In the long term, this technique may reduce the rate of revision and thus the cost of medical care. We need to further study cost-effective of robot-assisted TKA.

Perhaps the most important finding of our study is that the tourniquet in robot-assisted TKA does not significantly decrease total blood loss. While the tourniquet appears to reduce intraoperative blood loss and shorten the operation, it fails to decrease hidden blood loss. Similarly, a study involving conventional TKA [25] found that the tourniquet can effectively control intraoperative blood loss but not reduce postoperative or total blood loss. On the contrary, a randomized controlled trial suggested that tourniquet use during conventional TKA significantly decreases blood loss without adversely affecting early postoperative outcomes [26]. In a meta-analysis including 11 RCTs, Cai et al. [21] demonstrated that tourniquet use did not significantly decrease postoperative blood loss and total blood loss. However, there is no meta-analysis report on the effect of tourniquet in robot-assisted TKA on the blood loss. We may need a high-quality meta-analysis to assess whether the tourniquet can significantly reduce total blood loss in robot-assisted TKA.

Whatever the case, it seems clear enough that using a tourniquet increases hidden blood loss [27]. Hidden blood loss arising from postoperative hyperfibrinolysis accumulates in the third anatomic space and has been associated with postoperative inflammation, lower limb swelling, subcutaneous ecchymosis, and pain [28]. Our swelling data are consistent with this. Since swelling is associated with quadriceps weakness and slower gait [29], interventions to reduce knee swelling after TKA may strengthen quadriceps and improve gait speed.

Numerous studies have shown that using a tourniquet during conventional TKA does not reduce total blood loss, but instead it appears to increase risk of more severe pain, longer length of stay, slower functional recovery, and complications [21, 26, 30–32], similar to our results. Since robot-assisted TKA takes significantly longer than conventional surgery [33], and since risk of tourniquet-associated complications increases with tourniquet time [34], we suspect that at least some of the adverse effects of tourniquet use in robot-assisted TKA relate to the relatively long operation time.

Our study demonstrated that tourniquets are associated with more severe pain soon after robot-assisted TKA, especially pain in the thigh. In fact, the pain appears to be higher than that reported for tourniquet use during conventional TKA [27]. Pain in the early postoperative period can strongly affect outcomes because it can compromise functional recovery [35]. Using a tourniquet can cause pain due to ischemia–reperfusion injury [36], particular when the tourniquet is used longer. The use of registration pins in robot-assisted TKA increases pain even more.

We found that range of motion on PODs 1–3 was significantly restricted when a tourniquet was used, which is consistent with a report that tourniquets are slow functional recovery after routine TKA [37]. Indeed, we found that using a tourniquet in robot-assisted TKA led to longer time to achieve the first straight-leg raise than what has been reported for patients after conventional TKA [38], which likely reflects muscle damage as a result of prolonged tourniquet use. Indeed, the ischemia–reperfusion injury associated with tourniquets may significantly reduce the number of skeletal muscle fibers [39]. Bulkley has suggested that superoxide produced from xanthine oxidase in the reperfused vascular endothelial cell causes upregulation of adhesion molecules on the luminal surface of the endothelial cell [40]. These molecules react with complementary ligands on circulating neutrophils, which are consequently arrested and activated releasing damaging proteases and oxidants [40]. With a tourniquet may damage quadriceps, thereby delaying the recovery of muscle function, prolonging hospitalization, and giving rise to long-lasting functional deficits [41]. However, we found no significant difference between the tourniquet and non-tourniquet groups in functional recovery up to 3 months after robot-assisted TKA. Studies with longer follow-up should verify and extend our results.

Compared with conventional surgery reported in previous data [38], we found that tourniquet use in robot-assisted TKA resulted in a higher incidence of blisters and DVT, which we believe may be due to the prolonged tourniquet use, which we believe may be explained by the excessive duration of tourniquet use. The use of a pneumatic tourniquet in conventional TKA may increase risk of postoperative complications [42, 43], although there is some evidence that it can protect against DVT [44, 45]. We found that a tourniquet significantly increased the risk of tension blisters but not of other complications, even DVT. More work is needed to clarify the effects of tourniquet use on DVT and other complications after TKA, especially since the literature shows substantial heterogeneity of samples and study designs [27]. Clarifying these effects is particularly important for robot-assisted TKA because it involves longer operation and tourniquet time than routine TKA.

One of the strengths of our study is that it is a randomized, controlled, double-blinded trial. Another advantage is that all patients received the same standardized perioperative treatment, including multimodal analgesia, surgical procedure, and blood loss management. This should reduce heterogeneity that could confound our analysis. On the other hand, our study was small and did not examine long-term clinical outcomes. The small sample may have failed to detect significant effects of a tourniquet on the incidence of complications, especially the DVT and pulmonary embolism. Large, multi-center studies are needed to evaluate the safety and efficacy of tourniquet use in robot-assisted TKA, especially now that robot-assisted surgery is becoming popular.

## Conclusion

Using a tourniquet in robot-assisted TKA does not appear to reduce total or hidden blood loss, but instead it appears to increase risk of more severe pain, longer length of stay, slower functional recovery, and complications. Therefore, we do not recommend tourniquets in robot-assisted TKA.

## Data Availability

Not applicable.

## Abbreviations

TKA: Total knee arthroplasty
VAS: Visual analog scale
BMI: Body mass index
APTT: Activated partial thromboplastin time
PT: Prothrombin time
FIB: Fibrinogen
TT: Thrombin time
CT: Computed tomography
TXA: Tranexamic acid
HSS: Hospital for Special Surgery
PODs: Postoperative days
DVT: Deep vein thrombosis
Fig: Figure
